# Correlations between radiographic, magnetic resonance and histological examinations on the degeneration of human lumbar intervertebral discs

**DOI:** 10.1590/S1516-31802010000200004

**Published:** 2010-03-04

**Authors:** Delio Eulalio Martins, Valdeci Manoel de Oliveira, Maria Teresa de Seixas Alves, Marcelo Wajchenberg, Élcio Landim, João Carlos Belloti, Eduardo Barros Puertas, Akira Ishida

**Affiliations:** I MD. Attending physician in the Spine Group, Department of Orthopedics and Traumatology, Universidade Federal de São Paulo (Unifesp), São Paulo, Brazil.; II MD, PhD. Adjunct professor of Anatomy, Faculdade de Ciências Médicas e da Saúde de Juiz de Fora (FCMS/JF), and deputy professor of Orthopedics and Traumatology, School of Medicine, Universidade Federal Juiz de Fora (UFJF), Juiz de Fora, Minas Gerais, Brazil.; III MD, PhD. Adjunct professor and head of General Pathological Anatomy, Forensics and Bioethics, Department of Pathology, Universidade Federal de São Paulo (Unifesp), São Paulo, Brazil.; IV Brazil MD, MSc. Attending physician in the Spine Group, Department of Orthopedics and Traumatology, Universidade Federal de São Paulo (Unifesp), São Paulo, Brazil.; V Brazil MD, PhD. Assistant professor, Department of Orthopedics and Traumatology, Faculdade de Ciências Médicas da Santa Casa de São Paulo, and head of Scoliosis Group, Associação de Assistência à Criança Deficiente (AACD) de São Paulo, São Paulo, Brazil.; VI MD, PhD. Doctor of Science, associate professor, Department of Orthopedics and Traumatology, Universidade Federal de São Paulo, São Paulo, Brazil.; VII MD, PhD. Associate professor and head of Traumatology, Department of Orthopedics and Traumatology, Universidade Federal de São Paulo (Unifesp), São Paulo, Brazil.; VIII MD, PhD. Full professor, Department of Orthopedics and Traumatology, Universidade Federal de São Paulo (Unifesp), São Paulo, Brazil.

**Keywords:** Intervertebral disk, Spine, Magnetic resonance spectroscopy, Radiography, Nerve endings, Comparative study [Publication type], Disco intervertebral, Coluna vertebral, Espectroscopia de ressonância magnética, Radiografia, Terminações nervosas, Estudo comparativo [Tipo de publicação]

## Abstract

**Context And Objective:**

There is controversy regarding which imaging method is best for identifying early degenerative alterations in intervertebral discs. No correlations between such methods and histological finds are presented in the literature. The aim of this study was to correlate the thickness of intervertebral discs measured on simple radiographs with the degree of degeneration seen on magnetic resonance images and the histological findings relating to nerve ends inside the discs.

**Design And Setting:**

Cross-sectional correlation study on the lumbar spines of human cadavers, at Universidade Federal de São Paulo (Unifesp), São Paulo, Brazil.

**Methods:**

Ten lumbar spinal columns were extracted from human cadavers and subjected to magnetic resonance imaging and simple radiography. They were classified according to the degree of disc degeneration seen on magnetic resonance, and the thickness of the discs was measured on radiographs. The intervertebral discs were then extracted, embedded in paraffin and analyzed immunohistochemically with protein S100, and the nerve fibers were counted and classified.

**Results:**

No correlation was observed between the thickness of the intervertebral discs and the degree of degeneration seen on magnetic resonance images. Only the uppermost lumbar discs (L1/L2 and L2/L3) presented a correlation between their thickness and type I and IV nerve endings.

**Conclusion:**

Reduced disc thickness is unrelated to increased presence of nerve ends in intervertebral discs, or to the degree of disc degeneration.

## RESUMO

**Contexto E Objetivo:**

Há controvérsia sobre qual o melhor método de imagem para identificar alterações degenerativas precoces do disco intervertebral. Falta na literatura correlação desses métodos com os achados histológicos. O objetivo deste estudo foi relacionar a altura dos discos intervertebrais medidos em radiografias simples com o grau de degeneração nas imagens de ressonância magnética e os achados histológicos das terminações nervosas encontradas no interior do disco.

**Tipo De Estudo E Local:**

Estudo transversal de correlação em coluna lombar de cadáveres humanos, na Universidade Federal de São Paulo (Unifesp), São Paulo, Brasil.

**Métodos:**

Dez colunas lombares foram retiradas de cadáveres humanos e submetidas a imagens de ressonância magnética e radiografias simples. Foram classificadas de acordo com o grau de degeneração dos discos pela ressonância e mensuradas as alturas dos discos nas radiografias. Os discos intervertebrais foram retirados, incluídos em parafina e foi realizado estudo imunoistoquímico com proteína S100; as fibras nervosas foram contadas e classificadas.

**Resultados:**

Não foi observada correlação entre a altura dos discos intervertebrais com o grau de degeneração nas imagens de ressonância magnética. Apenas os discos lombares altos (L1/L2 e L2/L3) apresentaram correlação entre a altura e as terminações nervosas dos tipos I e IV.

**Conclusão:**

A diminuição da altura dos discos não está relacionada ao aumento de terminações nervosas nos discos intervertebrais e nem com o grau de degeneração dos discos.

## INTRODUCTION

Lumbar pain is one of the most frequent causes of time off work among adults of productive age in developed countries.^1,2^ Degenerative disc disease (DDD) is among the most prevalent causes of lumbar pain.^1^ However, it should be emphasized that these two terms are not synonymous,^3^ even though pain severity has been correlated with the start of DDD symptoms.^4,5^

During the process of intervertebral disc degeneration, losses of water, proteoglycan and collagen content from inside the disc occur. These can be noted on magnetic resonance images with T2 weighting as decreased signal intensity, and on radiographs as a loss of thickness of the intervertebral discs.^1,6^

Degeneration of the intervertebral discs has been studied and classified by many authors.^7-13^ The classifications range from studies of purely histological nature on the discs^10^ and on variations in their endplates,^7^ to classifications of abnormalities of the nucleus pulposus in relation to the annulus fibrosus and the thickness of the discs seen on magnetic resonance images.^8,11^

The thickness of intervertebral discs also seems to be correlated with early alterations in the discs. It can be measured in various ways, by means of standard radiography in the sagittal plane.^13-15^ Disc thickness seems to be one of the best parameters for correlations with morphological abnormalities in the discs.^1^ However, we did not find any data in the literature correlating the thickness with the nerve ends present in degenerated discs.

Degenerated intervertebral discs present a greater concentration of vessels and nerve ends, located particularly in the external third of the annulus fibrosus^16-18^ and anterior longitudinal ligament.^17,18^ However, the clinical implications of these findings and their correlations with abnormalities of the intervertebral discs remain controversial.

## OBJECTIVE

The aim of the present study was to correlate the thickness of the intervertebral discs on simple radiographs, the abnormalities presented on magnetic resonance images and the quantitative and qualitative variations in nerve ends that are seen in histological findings from the lumbar intervertebral discs of humans.

## MATERIAL AND METHODS

Ten lumbar spinal columns were extracted from cadavers of mean age 51 years (range from 30 to 85 years), of which five were male and five were female. The whole lumbar spine, from L1 to L5, was removed during the necropsy at the coroner’s office (Serviço de Verificação de Óbitos da Capital) of the School of Medicine, Universidade de São Paulo (USP), after approval had been obtained from the local Ethics Committee of Universidade Federal de São Paulo (Unifesp). Donors with any pathological condition directly affecting the vertebral column, such as tumors or fractures, were excluded from this study.

The specimens were subjected to magnetic resonance examinations using Philips ACS-NT apparatus, of 1.5 tesla. Images with T1 weighting (repetition time 452 and echo time 14) and T2 weighting (repetition time 3367 and echo time 140) were produced. The sections were 4 mm in thickness, with a field of view (FOV) of 27 cm. The images were produced at Unifesp. The images were evaluated and classified by a radiologist with proven experience, in accordance with the Pfirrmann et al.^8^ classification.

**Figure 1.**
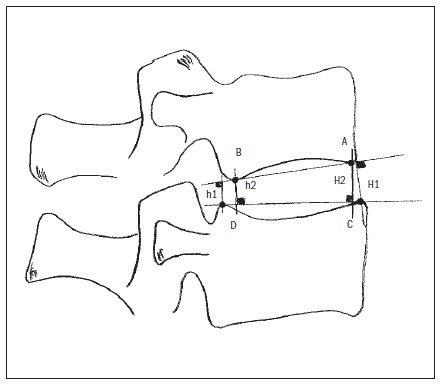
Measurement of disc height according to a method modified from Farfan.^14^ Disc height = (h1 + h2 + H1 + H2)/4. A: anteroinferior corner of the upper vertebral body; B: posteroinferior corner of the upper vertebral body; C: anterosuperior corner of the lower vertebral body; D: posterosuperior corner of the lower vertebral body.

Following this, the spinal columns were subjected to standard radiography using the anteroposterior and sagittal views, with 55/16 kV, using Med X-50 F apparatus (No. 001-01; 50/60 Hz; 380/220 V). The images were digitized and, using the Image Pro-Plus^□^ software, the thickness of the intervertebral discs was measured by means of a method modified from Farfan^13-15,19^ as in **Figure 1**.

The spinal columns were prepared no more than 48 hours after death, in order to minimize any changes to the disc matrix. The whole spines were kept in ice, inside dried sealed bags, while being transported to the radiological center, thereby minimizing the water loss and preserving the disc matrix characteristics. The discs were fixed in 10% formalin, dehydrated in increasing concentrations of alcohol, diaphanized in xylol and embedded in paraffin. The paraffin blocks were cooled and histological sections of 3 μm to 4 μm in thickness were cut. These paraffinized sections were placed on slides that had previously been treated with 3-aminopropyltriethoxysilane (APTS). The slides were then incubated with the primary antibody (protein S100), diluted in bovine albumin. Hematoxylin was used for counterstaining.

**Figure 2.**
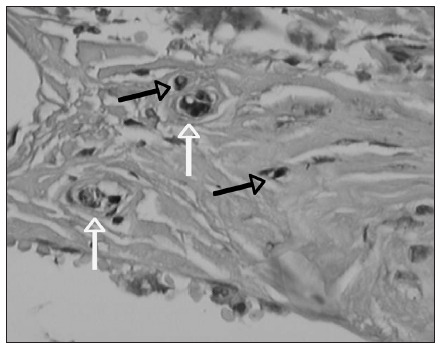
Photomicrograph of intervertebral disc stained by means of an immunohistochemical method for protein S100. Magnification 400 x. Black arrows demonstrate type I fibers; white arrows demonstrate type II fibers.

**Figure 3.**
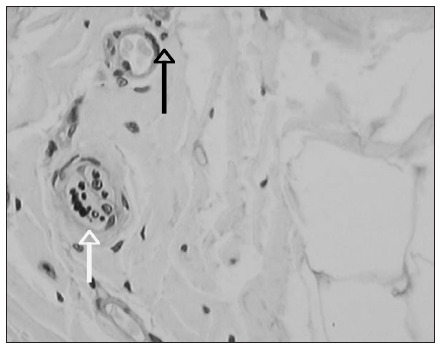
Photomicrograph of intervertebral disc stained by means of an immunohistochemical method for protein S100. Magnification 400 x. The white arrow demonstrates a type III fiber, stained brown. A Type IV fiber (black arrow), at the periphery of a blood vessel can also be seen.

Following this, the nerve fibers were counted over the whole extent of the disc, at 400 x magnification, with the aid of a video camera (JVC model TK 1180V). This transmitted the image captured, from the microscope (Olympus model BX40) to a Pentium MMX 233 MHz computer equipped with a digitizing board and the Image Pro-Plus^□^ software version 6.3 in the Windows^□^ environment. The fibers were measured and classified according to size and shape, using the classification system of Freeman and Wyke^20^ (**Figures 2 and 3**).

Using the Statistical Package for the Social Sciences (SPSS) for Windows 8.0, the correlations between the variables were evaluated by means of the Spearman rank correlation coefficient (rs), and its significance was tested. The significance level was taken to be 0.05 (α = 5%), and descriptive levels (P) less than this value were considered significant.

## RESULTS

The variables of thickness and number of nerve fibers were summarized and represented by the mean, standard deviation (SD), median, minimum and maximum. The degree of degeneration was summarized and represented by absolute and relative (%) frequencies.

We did not find any type 5 Pfirrmann intervertebral discs in this sample. In two cases, the discs were intermediate between two degrees of degeneration and therefore we took the greater degree. There was no correlation between the thickness and the degree of degeneration of the intervertebral discs (P > 0.05).

Freeman type I to IV nerve endings were found in all the discs studied, in all disc regions and at the different degrees of degeneration.

**Figure 4.**
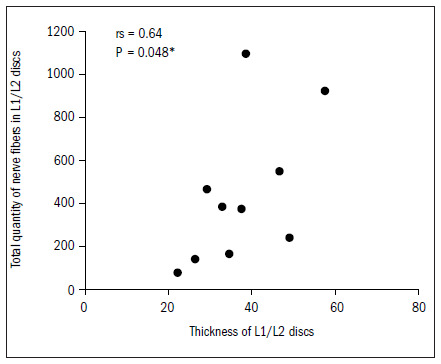
Relationship between thickness and total quantity of nerve fibers in L1/L2 discs.

**Figure 5.**
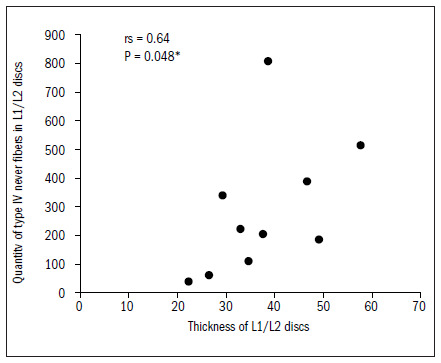
Relationship between thickness and quantity of type IV nerve fibers in L1/L2 discs.

The L1/L2 intervertebral discs presented a significant correlation between disc thickness and the quantities of nerve fibers in total and of Freeman type IV endings (P < 0.05), as demonstrated in **Figures 4** and **5**. For the L2/L3 discs, there were statistical correlations with type I and IV and with the total quantity (P < 0.05) and marginally significant correlations with type II and III fibers (0.05 < P < 0.10).

**Figure 6.**
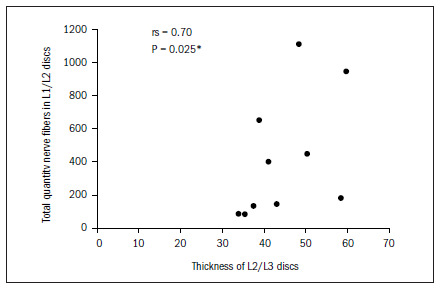
Relationship between thickness and total quantity of nerve fibers in L2/L3 discs.

**Figure 7.**
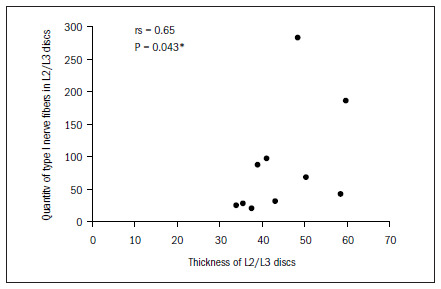
Relationship between thickness and quantity of type I nerve fibers in L2/L3 discs.

**Figure 8.**
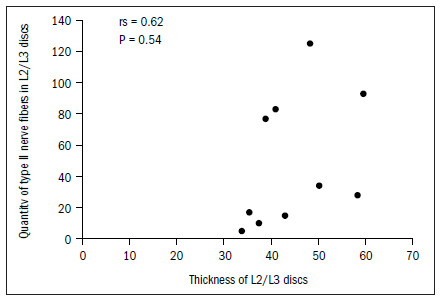
Relationship between thickness and quantity of type II nerve fibers in L2/L3 discs.

**Figure 9.**
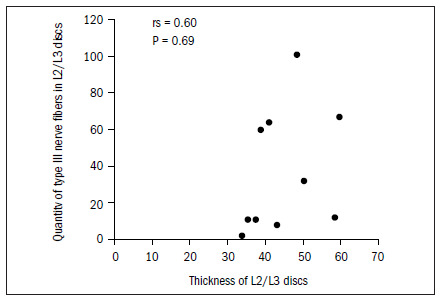
Relationship between thickness and quantity of type III fibers in L2/L3 discs.

**Figure 10.**
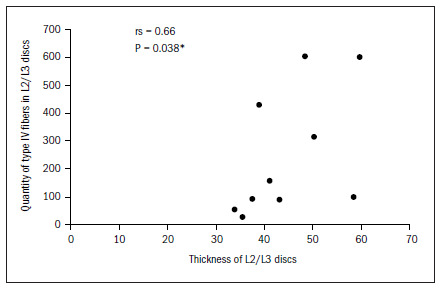
Relationship between thickness and quantity of type IV fibers in L2/L3 discs.

The data demonstrated that the greater the thickness of the L1/L2 and L2/L3 discs was, the greater the total quantity of nerve fibers was, and particularly the quantity of type IV. In the L2/L3 discs, there was also a correlation with type I fibers. There was a tendency (not shown statistically) towards a correlation between greater thickness of this disc and greater quantities of type II and type III fibers, as shown in **Figures 6** to **10**. In all other discs, the coefficients between thickness and quantity of nerve fibers were nonsignificant (P > 0.05).

## DISCUSSION

The degeneration of intervertebral discs has been extensively studied but, despite the many advances achieved, there is still little knowledge of the exact cause of lumbar pain and of the correlation between such degeneration and the currently used imaging investigation methods. The sample could not be calculated with precision because the lack of data in the literature comparing the heights of intervertebral discs to findings from MRI. However, we examined around 30% more discs (50 against 39) than in one of the best previous studies in this field.^1^ There was not even any precise definition of degeneration until Adams and Roughley^21^ recently proposed the following definition: "disc degeneration is an aberrant cell-mediated response to progressive failure of the disc structure".

Several methods for attempting to assess disc degeneration are known. The method for measuring intervertebral discs described by Farfan^19^ continues to be one of the most reliable ways of measuring the thickness of the intervertebral space. However, it uses the disc diameter as the denominator for correcting the magnification, which makes this an equation that correlates more with disc shape than with disc thickness. Thus, in the present study, we observed the thickness of 50 intervertebral discs, measured in accordance with the Farfan^19^ method, as modified by Dabbs’^14^ (**Figure 1**). In this method, the mean from the anterior and posterior thicknesses of the disc was obtained, thus giving a more trustworthy value for disc thickness.

Several classification methods have been used for disc degeneration, going from methods using exclusively histological parameters^10^ to classifications that attempt clinical correlation through describing the discs using magnetic resonance and radiography.^12^ The disc degeneration was classified as described by Pfirrmann et al,^8^ since this presents excellent intra and interobserver reliability. Benneker et al.^1^ correlated the findings relating to magnetic resonance, simple radiography, biochemical abnormalities and morphological abnormalities from 39 intervertebral discs from cadavers. They concluded that the radiographic parameters (particularly the thickness of the intervertebral discs) correlated better with the different stages of degeneration than did the magnetic resonance images alone. In our sample, we did not find any correlation between disc thickness and the degree of degeneration, which corroborates the findings of other authors such as Schiebler et al.^22^ These authors studied early degeneration of intervertebral discs in live patients and in cadavers and concluded that magnetic resonance showed early changes in intervertebral discs of normal thickness. Moreover, Tertti et al.^23^ studied 89 intervertebral discs from 22 lumbar spinal columns from cadavers and correlated the signal intensity findings from magnetic resonance with the findings relating to biochemical composition, conventional radiography and histological structure. They concluded that the low signal intensity in images of the lumbar spine with T2 weighting represented biochemical degeneration of the disc, but that the correlation with degenerative alterations to disc structure was uncertain. Furthermore, Videman et al.^24^ retrospectively studied 115 pairs of monozygotic twins (all of them male), by means of a detailed questionnaire on their histories of lumbar pain and by means of magnetic resonance examinations, in which the thickness of the intervertebral disc, protrusions, disc herniations, fissures of the annulus, osteophytes, spinal stenosis and endplate abnormalities were evaluated. They concluded that the disc thickness seen on magnetic resonance and the fissures of the annulus presented poor sensitivity and were of little clinical importance.

With regard to the presence of nerve fibers in the intervertebral discs, there is a vast body of literature demonstrating that these fibers are located in the external third of the annulus fibrosus and that they do not reach the internal portion of the disc. Roberts et al.^17,18^ studied the intervertebral discs of bovines and humans to investigate the distribution and morphology of the mechanical receptors present in this region, and to correlate this with lumbar pain and scoliosis. These authors concluded that the mechanical receptors were present in the external part of the annulus fibrosus of the intervertebral disc and in the anterior longitudinal ligament, in humans. Oliveira et al.^16^ studied the nerve ends that exist in the human lumbar spine, using five columns from young adult cadavers. They found nerve ends over the whole external surface and in the superficial layer of the annulus fibrosus. They did not find nerve ends in the internal layer of the annulus fibrosus or in the nucleus pulposus.

In our study, there was a correlation between the thickness of the intervertebral discs and the quantity and type of nerve ends. In the L1/L2 discs, the correlation was inversely positive for Freeman and Wyke^20^ type IV endings, i.e. the greater the thickness was, the greater the quantity of type IV nerve ends apparently relating to nociception was. In the L2/L3 discs, the correlation was also inversely positive for the type I nerve ends (mechanical receptors) and type IV nerve ends. For the other discs, we did not obtain any type of correlation. Roberts et al.^17^ found greater incidence of nerve ends in the discs of patients with lumbar pain than in the discs of pain-free patients and those with scoliosis. However, they could not confirm that the mechanical receptors were related to pain, because of the heterogeneity of the groups and also because nerve ends were present in only 50% of the discs.

## CONCLUSIONS

In our study, it was impossible to make a clinical correlation from the findings because the study was conducted on cadavers. Nonetheless, from the results found, we can conclude that decreased disc thickness was unrelated to increased presence of nerve ends and to abnormalities on magnetic resonance images of intervertebral lumbar discs in humans. This decreased thickness is probably only related to biochemical changes and loss of hydration in the discs, and further studies must be conducted to analyze this.

## References

[1] Benneker LM, Heini PF, Anderson SE, Alini M, Ito K (2005). Correlation of radiographic and MRI parameters to morphological and biochemical assessment of intervertebral disc degeneration. Eur Spine J.

[2] Andersson GB (1998). Epidemiology of low back pain. Acta Orthop Scand Suppl.

[3] Battié MC, Videman T, Parent E (2004). Lumbar disc degeneration: epidemiology and genetic influences. Spine (Phila Pa 1976).

[4] Peterson CK, Bolton JE, Wood AR (2000). A cross-sectional study correlating lumbar spine degeneration with disability and pain. Spine (Phila Pa 1976).

[5] Luoma K, Riihimäki H, Luukkonen R, Raininko R, Viikari-Juntura E, Lamminen A (2000). Low back pain in relation to lumbar disc degeneration. Spine (Phila Pa 1976).

[6] Urban JPG, Roberts S, Herkowitz H, Garfin SR, Eismont FJ, Bell GR, Balderston RA (2006). The intervertebral disc: normal, aging and pathologic. Rothman-Simeone the spine.

[7] Modic MT, Masaryk TJ, Ross JS, Carter JR (1988). Imaging of degenerative disk disease. Radiology.

[8] Pfirrmann CW, Metzdorf A, Zanetti M, Hodler J, Boss N (2001). Magnetic resonance classification of lumbar intervertebral disc degeneration. Spine (Phila Pa 1976).

[9] Southern EP, Fye MA, Panjabi MM, Patel TC, Cholewicki J (2000). Disc degeneration: a human cadaveric study correlating magnetic resonance imaging and quantitative discomanometry. Spine (Phila Pa 1976).

[10] Thompson JP, Pearce RH, Schechter MT, Adams ME, Tsang IK, Bishop PB (1990). Preliminary evaluation of a scheme for grading the gross morphology of the human intervertebral disc. Spine (Phila Pa 1976).

[11] Griffith JF, Wang YX, Antonio GE (2007). Modified Pfirrmann grading system for lumbar intervertebral disc degeneration. Spine (Phila Pa 1976).

[12] Thalgott JS, Albert TJ, Vaccaro AR (2004). A new classification system for degenerative disc disease of the lumbar spine based on magnetic resonance imaging, provocative discography, plain radiographs and anatomic considerations. Spine J.

[13] Rillardon L, Campana S, Mitton D, Skalli W, Feydy A (2005). Analyse de l’espace intervertébral avec un système de radiographie basse dose [Evaluation of the intervertebral disc spaces with a low dose radiographic system]. J Radiol.

[14] Dabbs VM, Dabbs LG (1990). Correlation between disc height narrowing and low-back pain. Spine (Phila Pa 1976).

[15] Pope MH, Hanley E, Matteri RE, Wilder DG, Frymoyer JW (1977). Measurement of intervertebral disc space height. Spine.

[16] Oliveira VM, Puertas EB, Alves MTS (2002). Estudo das terminações nervosas dos discos intervertebrais da coluna lombar de humanos [Study of nerve endings of intervertebral discs in the human lumbar spine]. Rev Bras Ortop.

[17] Roberts S, Eisenstein SM, Menage J, Evans EH, Ashton IK (1995). Mechanoreceptors in intervertebral discs Morphology, distribution, and neuropeptides. Spine (Phila Pa 1976).

[18] Roberts S, Evans H, Trivedi J, Menage J (2006). Histology and pathology of the human intervertebral disc. J Bone Joint Surg Am.

[19] Farfan HF (1973). Mechanical disorders of the low back.

[20] Freeman MA, Wyke B (1967). The innervation of the knee joint. An anatomical and histological study in the cat. J Anat.

[21] Adams MA, Roughley PJ (2006). What is intervertebral disc degeneration, and what causes it?. Spine (Phila Pa 1976).

[22] Schiebler ML, Camerino VJ, Fallon MD, Zlatkin MB, Grenier N, Kressel HY (1991). In vivo and ex vivo magnetic resonance imaging evaluation of early disc degeneration with histopathologic correlation. Spine (Phila Pa 1976).

[23] Tertti M, Paajanen H, Laato M, Aho H, Komu M, Kormano M (1991). Disc degeneration in magnetic resonance imaging A comparative biochemical, histologic, and radiologic study in cadaver spines. Spine (Phila Pa 1976).

[24] Videman T, Battié MC, Gibbons LE, Maravilla K, Manninen H, Kaprio J (2003). Associations between back pain history and lumbar MRI findings. Spine (Phila Pa 1976).

